# Dr. Rosting

**Published:** 1852-04

**Authors:** 


Dr. Rosting who formerly practised dentistry in this city on Fourth street, is now in Dresden, and getting along well in his profession judging from the following, which we find in a foreign letter published in the *Catholic Telegraph* of this city, dated Dresden, July 7, 1851."

"We had the pleasure of meeting Dr. Rosting and his family, who are now residing here and doing well. He is the dentist to the Grand Duke of Saxe Weimar, and also to some part of the royal family of the King of Prussia at Berlin, and the royal family of Saxony here. He has been knighted and moves here in the highest circles, but says he is still an American and a true Democrat."

The above we received from a friend, and know not from what paper it was clipped.

We are glad to hear that our old friend Dr. Rosting is distinguishing himself among the distinguished of the earth, and hope he may find it not only pleasant but profitable.—Ed.

